# Impact of cigarette smoking on the relationship between body mass index and coronary heart disease: a pooled analysis of 3264 stroke and 2706 CHD events in 378579 individuals in the Asia Pacific region

**DOI:** 10.1186/1471-2458-9-294

**Published:** 2009-08-13

**Authors:** The Asia Pacific Cohort Studies Collaboration

**Affiliations:** 1 Nutrition and Lifestyle Division, The George Institute for International Health, Missenden Road, Camperdown, Australia

## Abstract

**Background:**

Elevated levels of body mass index (BMI) and smoking are well established lifestyle risk factors for coronary heart disease (CHD) and stroke. If these two risk factors have a synergistic relationship, rigorous lifestyle modification may contribute to greater reduction in cardiovascular burden than previously expected.

**Methods:**

A pooled analysis of individual participant data from 38 cohorts, involving 378,579 participants. Hazards ratios (HRs) and 95% confidence intervals (CIs) for BMI by cigarette smoking status were estimated using Cox proportional hazard models.

**Results:**

During a mean follow-up of 3.8 years, 2706 CHD and 3264 strokes were recorded. There was a log-linear, positive relationship of BMI with CHD and stroke in both smokers and non-smokers with evidence of a synergistic effect of smoking on the association between BMI and CHD only: HRs (95% CIs) associated with a 2 kg/m^2 ^higher BMI were 1.13 (1.10 – 1.17) in current smokers and 1.09 (1.06 – 1.11) in non-smokers (p-value for interaction = 0.04).

**Conclusion:**

Smoking amplifies the positive association between BMI and CHD but not stroke. If confirmed, these results suggest that effective strategies that target smoking cessation and weight loss are likely to have a greater impact than anticipated on reducing the burden of CHD.

## Background

It has been well documented that overweight and obesity is now a global epidemic with relatively few countries unaffected. The World Health Organisation (WHO) has estimated that 1.6 billion people are overweight worldwide, which, for the first time, exceeds those who are undernourished [1,2]. Moreover, low- and middle-income countries, such as China that traditionally had one of the leanest populations, are rapidly approaching the prevalence of overweight observed in more developed regions [3]. Although the prevalence of smoking in the Western world has progressively declined over the past forty years – due largely to effective and comprehensive tobacco control measures – tobacco consumption remains high in many countries across Asia where about 40–70% of men smoke [4]. This is a likely consequence of the low level of awareness of the health hazards associated with smoking combined with a low use of smoking cessation aids and aggressive promotion of smoking by the tobacco industry in many countries in Asia, such as China [5].

Smoking [6-8] and excess weight [9-11] are well established risk factors for cardiovascular disease. In smokers, the risk of coronary heart disease (CHD) and stroke is more than twice that of non-smokers [7], whereas there is a somewhat more modest increase in risk associated with overweight and obesity [11]. Some previous studies have suggested that the association between body mass index (BMI) and cardiovascular risk may be modified by smoking, although there is dispute over whether smoking amplifies or attenuates the relationship [12-15]. If smoking does interact with BMI, this would have ramifications for estimates of disease burden due to these two important and modifiable risk factors, and have implications for disease prevention. In the present study, we take advantage of the Asia Pacific Cohort Studies Collaboration (APCSC) – a large database comprising more than 40 longitudinal studies from the region – to explore this important public health issue.

## Methods

### Participating studies

The APCSC is an individual participant data overview conducted by the principal investigators of 44 existing cohort studies in the region. Methods of study identification, their characteristics have been reported elsewhere [16,17]. In brief, studies were eligible for inclusion in the project if they satisfied the following criteria: (1) a study population from the Asia Pacific region; (2) prospective study design; (3) at least 5,000 person-years of follow-up recorded; (4) date of birth (or age), sex, and blood pressure recorded at baseline; (5) date of death (or age at death) recorded during follow-up. Additionally, for analyses in this report, participants were restricted to individuals aged 20 years or over at study entry with information on both BMI and smoking status.

### Measurement of baseline variables

Height and weight were measured using standard protocols when the participant entered the study, and BMI was calculated as weight (kg) divided by squared height (m^2^). Cigarette smoking status (current smoker/non-smoker) at baseline was assessed on the basis of self-report. Whether an individual was a current smoker, a former smoker or a never smoker was also reported in a smaller number of studies. Cohorts were classified as Asian if the participants were recruited from Mainland China, Hong Kong, Japan, Korea, Singapore, Taiwan or Thailand and as Australasian (ANZ) if the participants were from Australia or New Zealand. This classification largely represented a split by ethnicity into Asians and non-Asians.

### Outcomes

Outcomes included in this report were total (fatal and non-fatal) coronary heart disease (CHD), total ischaemic stroke, total haemorrhagic stroke (primary intracerebral haemorrhage and subarachnoid haemorrhage) and unclassified stroke. Non-fatal events were defined as those that did not result in death within 28 days. Outcomes were coded according to the Ninth Revision of the International Classification of Diseases (ICD-9): Combined fatal and non-fatal CHD events (ICD-9: 410 – 414) and stroke events [total stroke (ICD-9: 430–438); haemorrhagic stroke (431.0–432.9); and ischaemic stroke (433.0–434.9)]. Since most studies used record linkage with official sources, verification of strokes was not routinely reported. However, 12 of the 36 studies included in this analysis provided information on stroke verification, and in 6 of them (n = 155252) a pathological subtype of stroke was determined by CT/MRI scan or brain autopsy in over 75% of stroke cases (n = 2448). All data provided to the Secretariat were checked for completeness and consistency and re-coded where necessary to maximise comparability across cohorts. Summary reports were referred back to principal investigators of each collaborating study for review and confirmation.

### Statistical methods

Participants at extreme ends of the BMI spectrum (i.e., <15 or >50 kg/m^2^) were excluded from the analysis (n = 303). To address the possibility of reverse causality whereby a low BMI may be indicative of some occult disease, all HRs (95% Confidence Intervals [95% CI]) reported in the results are derived after three years left-censoring of the data. Having ascertained that the proportional hazards assumption had not been violated, Cox proportional hazards regression models were used to regress time until first CHD or stroke event against levels of baseline BMI by smoking status (current smoker or non-smoker) [18]. All analyses were adjusted for age and stratified by study and sex. Further, to consider the potential effects of smoking on the relationships between BMI, CHD and stroke, independent of intermediary variables along the causal pathway between adiposity and cardiovascular outcomes we additionally adjusted for systolic blood pressure (SBP). We repeated these analyses within the sub-group of participants on whom information on all intermediary variables including SBP, serum total cholesterol (TC) and diabetic status were available. To explore log-linearity of the associations between BMI and CHD and stroke events, HRs were plotted for mean level in each of five BMI categories (the penultimate lowest category was the referent). For HRs in each category, 95% CIs were estimated by the 'floating absolute risk' method [19].

HRs with 95% CIs were also calculated for a 2 kg/m^2 ^higher BMI as a continuous variable. Further HRs (95% CIs) were calculated comparing the effects of smoking status in individuals classified either as overweight (BMI ≥ 25 kg/m^2 ^in ANZ and BMI ≥ 24 kg/m^2 ^in Asia) or "normal" weight (BMI ≤ 25 kg/m^2 ^in ANZ and BMI ≤ 24 kg/m^2 ^in Asia [20]. The statistical significance of any interaction between BMI and smoking status was assessed using likelihood ratio tests, comparing the models with main effects only with the models that included the interaction term [21]. As well as analyses of the overall APCSC, pre-defined subgroup analyses were performed by sex, by region (Asia and ANZ) and by age at risk (restricted to the following age-groups: 35 – 59, 60 – 69, 70 – 79, 80 – 89 years).

Analyses were repeated on the sub-sample of the total population which had information on whether any non-smoker had previously smoked. In these sub-samples, participants were classified as 'current' if they smoked currently, 'never smokers' if they had never smoked and 'former smokers' if they had smoked but reported having already quit at study baseline. HRs for a 2 kg/m^2 ^higher level of BMI were estimated for each of these smoking groups, and compared using similar methods to the main analyses.

## Results

### Characteristics of the study population

Information on BMI and smoking status (current smoker or not) was available from 38 cohorts (86% of all studies in APCSC); 30 from Asia (Table 1). Overall, 378,579 participants were included in the analysis (76% Asians; 40% female) with a mean age of 47 years. Approximately one-third (34%) of study participants were classified as current smokers at baseline, with marked differences by sex and region: in Asia, 60% of men and 5% of women were current smokers, compared to 21% and 14%, respectively, in ANZ. In Asia, mean age and BMI were similar between smokers and non-smokers (46 versus 46 years; 22.8 versus 22.9 kg/m^2^), but in ANZ, current smokers were younger (47 years versus 53 years) and had a lower BMI (25.6 kg/m^2 ^versus 26.4 kg/m^2^) than non-smokers. Of the 38 cohorts included in the main analyses, 32 (24 in Asia) recorded former smoking status for non-smokers. Of 339,251 participants in these cohorts, approximately 17% were former smokers, 50% were never smokers and 32% were current smokers. In Asian cohorts, 23% of men and 23% of women who had ever smoked had quit, compared to 66% and 59%, respectively, in ANZ.

**Table 1:** Study population characteristics at baseline by smoking status

	Non-smokers						Current smokers					
		Age (years)		BMI (kg/m^2^)		Female		Age (years)		BMI (kg/m^2^)		Female
Study name	n	mean	SD	mean	SD	(%)	n	mean	SD	mean	SD	(%)

Akabane	1294	55	8	22.7	3.1	77	499	53	7	22.0	2.6	2
Anzhen	5778	54	13	24.1	3.7	70	2306	52	12	23.4	3.4	20
Anzhen02	2995	47	8	23.9	3.3	65	733	46	8	24.1	3.1	1
Beijing Aging	1281	69	8	24.0	3.9	63	545	68	8	22.4	3.7	24
Capital Iron & Steel Co.	1281	45	8	23.7	2.7	0	3570	45	8	23.0	2.7	0
CISCH	1535	44	7	24.6	3.5	70	572	45	8	24.6	3.1	2
Civil Service Workers	5614	47	5	22.5	2.7	47	3401	47	5	22.4	2.8	10
CVDFACTS	4188	47	15	23.5	3.4	71	1192	48	15	23.5	3.2	4
East Beijing	756	44	15	23.7	3.2	65	318	41	15	23.5	3.4	19
EGAT	1786	42	5	23.2	3.1	38	1315	43	5	22.8	3.1	3
Fangshan	842	47	11	24.6	3.4	87	556	48	10	23.2	3.3	37
Guangzhou Occupational	10534	42	6	22.8	3.0	61	9876	43	6	22.2	2.7	1
Hisayama	793	56	11	21.8	2.8	82	600	54	9	21.4	2.4	23
Hong Kong	637	78	6	22.5	3.7	74	164	75	5	21.1	3.4	40
Kinmen	121	67	11	23.5	3.5	67	56	67	10	22.4	4.1	18
KMIC	97831	44	7	22.9	2.5	54	60711	45	7	23.3	2.4	0
Konan	719	52	16	22.1	3.1	76	310	50	15	21.7	2.8	8
Miyama	688	60	9	22.4	3.0	74	297	60	9	21.7	2.7	13
Ohasama	1704	59	11	23.5	3.2	78	423	57	12	22.5	2.9	8
Saitama	2527	54	12	22.5	2.9	80	1001	54	12	22.0	2.8	17
Seven Cities Cohorts	3201	54	12	21.9	3.1	70	1809	53	12	20.8	2.8	22
Shibata	1472	56	11	22.8	3.2	83	730	56	10	21.8	2.6	8
Shigaraki Town	1742	58	14	22.8	3.1	77	711	56	13	21.8	2.8	13
Shirakawa	2989	48	12	21.6	2.8	79	1586	48	12	21.2	2.5	8
Singapore Heart	1768	40	13	23.8	4.4	61	495	41	14	22.8	4.0	7
Singapore NHS92	2649	39	12	23.3	4.1	62	594	38	12	22.9	4.2	8
Six Cohorts	10359	44	7	21.3	2.7	77	8804	45	7	21.1	2.4	12
Tanno/Soubetsu	1183	51	7	24.1	3.2	78	737	51	7	22.9	2.9	14
Tianjin	4254	55	12	24.4	4.0	65	4438	53	11	23.0	3.7	39
Yunnan	1979	57	10	22.0	3.0	10	4263	54	9	21.4	2.8	0
**Total Asia**	174500	46	10	22.9	2.9	59	112612	46	9	22.8	2.8	5

ALSA	871	77	6	26.3	4.0	51	60	76	5	24.9	4.3	52
ANHF	6932	44	13	25.5	4.3	53	2184	41	13	25.1	4.2	45
Busselton	4729	45	17	24.8	3.9	60	2450	43	16	24.2	3.6	37
Fletcher Challenge	7673	45	15	26.4	4.1	30	2362	40	12	26.5	4.3	22
Melbourne	36235	55	9	26.9	4.4	61	4561	53	8	26.6	4.3	48
Newcastle	4484	52	11	26.9	4.5	54	1324	49	10	25.9	4.3	41
Perth	7561	46	13	25.3	3.9	51	2569	43	12	24.7	3.8	40
WAAAAS	6707	72	4	26.9	3.6	0	765	71	4	26.0	4.0	0
**Total ANZ**	75192	53	14	26.4	4.3	50	16275	47	14	25.6	4.2	38

**Total**	249692	48	11	24.0	3.8	56	128887	46	10	23.1	3.1	9

BMI, body mass index; SD, standard deviation; ANZ, Australia and New Zealand; ALSA, Australian Longitudinal Study of Aging; ANHF, Australian National Heart Foundation; CISCH, Capital Iron and Steel Company Hospital; EGAT, Electricity Generating Authority of Thailand; KMIC, Korean Medical Insurance Corporation; NHS92, National Health Study 1992, WAAAAS, Western Australian AAA Screenees. Blanks indicate that the event was not reported for that study.

### Follow-up and outcomes

In total, there were 1,431,261 person-years of follow-up; the mean follow-up was 3.8 years (3.9 years for current smokers and 3.6 years for non-smokers) but it was shorter, for both current smokers and non-smokers, in Asia (2.9 and 2.8 years, respectively) than in ANZ (8.3 and 6.3 years) (Table 2). In addition to information on fatal events that was available from all cohorts, data on non-fatal events were present in 18 studies (Table 2). During follow-up there were 2706 CHD events (33% in Asia), of which myocardial infarction accounted for more than 80%, and 3264 stroke events (70% in Asia).

**Table 2:** Fatal and non-fatal coronary heart disease and stroke events by smoking status after three years left-censoring

	Number of events (female %)							
	
Region (Mean follow-up duration, years)	Non-smokers				Current smokers			
	
		Stroke sub-type				Stroke sub-type		
	CHD	Haem	Isch	NA	CHD	Haem	Isch	NA
Asia		272 (58)	180 (71)	445 (50)	262 (15)	268 (13)	165 (15)	458 (7)
(2.7 M, 3.1 F)		360 (53)	428 (59)	300 (65)	420 (12)	345 (11)	464 (10)	207 (8)
Fatal events	274 (59)							
Fatal and non-fatal events	369 (53)							
ANZ		64 (48)	49 (57)	511 (52)	451 (23)	26 (31)	12 (8)	265 (37)
(6.1 M, 7.5 F)		93 (50)	197 (52)	525 (54)	638 (24)	50 (34)	90 (34)	233 (37)
Fatal events	983 (40)							
Fatal and non-fatal events	1279 (40)							

**Total events**	**1648 (43)**	**453 (52)**	**625 (57)**	**825 (58)**	**1058 (20)**	**395 (14)**	**554 (14)**	**447 (24)**

ANZ = Australia and New Zealand; M = male; F = female; CHD = coronary heart disease; Haem = haemorrhagic stroke; Isch = ischaemic stroke; NA = unclassified stroke

**Figure 1:**
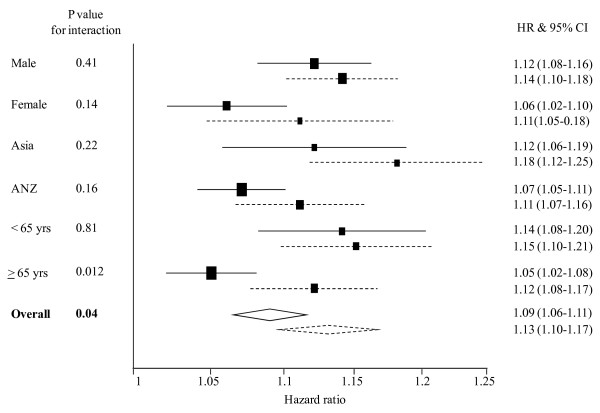
**Hazard ratios (HRs) associated with a 2 kg/m^2 ^higher body mass index for coronary heart disease events in current smokers and non-smokers, by sex, region, age and overall**. Analyses are adjusted by age and stratified by study and sex. The horizontal lines (or widths of diamonds for overall results) show 95% confidence intervals (CIs). The p values shown are for the test of interaction between body mass index and smoking status. The dashed and continuous lines represent current smokers and non-smokers, respectively.

The association between BMI and CHD in current and non-smokersHigher levels of BMI were positively and log-linearly associated with the risk of incurring a fatal or non-fatal CHD event, irrespective of smoking status (Table 3). There was strong evidence that compared with non-smokers, smoking amplified the strength of the association between BMI with CHD; this was apparent irrespective of whether BMI was dichotomised into "normal" and "overweight" or measured on a continuous scale. For example, current smokers who were overweight (25 < BMI ≤ 29.9 kg/m^2 ^in ANZ and 24 ≤ BMI ≤ 28 kg/m^2 ^in Asia) had approximately one-third greater excess risk of CHD compared with overweight (BMI > 30 kg/m^2 ^in ANZ and BMI > 28 kg/m^2 ^in Asia) non-smokers: HR 1.64 (95% CI: 1.47 – 1.82) vs. 1.47 (95% CI: 1.36 – 1.59); p-value for interaction = 0.019 (Table 3). Similarly, for a 2 kg/m^2 ^increment in BMI, the excess risk for CHD was greater in current smokers than in non-smokers by approximately four percentage points (i.e., 13% versus 9%) (Figure 1a): HR 1.13 (95% CI: 1.10 – 1.17) versus HR 1.09 (95% CI: 1.06 – 1.11); p-value for interaction = 0.04. When fatal CHD events alone (n = 1970) were analysed, similar patterns were observed: HR (95% CI) for a 2 kg/m^2 ^higher BMI was 1.14 (1.09 – 1.18) for current smokers and 1.09 (1.06 – 1.12) for non-smokers; p-value for interaction = 0.07.

**Table 3:** Adjusted hazard ratios (HRs) (with 95% confidence intervals) for the relationship between BMI with CHD in non- and current smokers in data that had been left-censored by three years

Smoking Status	BMI category (kg/m^2^)	Mean BMI (kg/m^2^)	No. of events	Age, sex, study adjusted HR for combined fatal and non-fatal CHD (95% CI)*	Age, sex, study adjusted HR (95% CI) for fatal CHD**
Non	15.0 – 18.4	17.6	46	1.31 (0.97 – 1.77)	1.34 (0.96 – 1.86)
	18.5 – 21.9	20.5	201	1.00 (0.87 – 1.15)	1.00 (0.85 – 1.18)
	22.0 – 24.9	23.4	457	1.34 (1.22 – 1.47)	1.40 (1.26 – 1.56)
	25.0 – 29.9	26.8	699	1.47 (1.36 – 1.59)	1.49 (1.36 – 1.63)
	30.0 – 50.0	33.1	245	1.83 (1.61 – 2.09)	1.90 (1.63 – 2.20)
					
Current	15.0 – 18.4	17.6	29	0.73 (0.50 – 1.06)	0.84 (0.56 – 1.24)
	18.5 – 21.9	20.5	237	1.00 (0.88 – 1.14)	1.00 (0.85 – 1.17)
	22.0 – 24.9	23.4	326	1.22 (1.10 – 1.36)	1.15 (1.01 – 1.32)
	25.0 – 29.9	26.6	362	1.64 (1.47 – 1.82)	1.49 (1.30 – 1.70)
	30.0 – 50.0	32.6	104	2.05 (1.68 – 2.51)	2.22 (1.76 – 2.79)

*P-value for interaction between never and current smokers = 0.019

** P-value for interaction between never and current smokers = 0.08

After adjustment for SBP the relationship between BMI and CHD was weakened but the interaction with smoking remained significant (p = 0.19). In the sub-group of individuals in whom data were available for SBP, TC and diabetes status (n = 318,384 and 2446 CHD events) there was no evidence of an interaction between smoking and BMI on subsequent risk of CHD before, or after, adjustment for these variables (p = 0.22 and p = 0.21, respectively), which is potentially due to the reduced power to detect any interaction.

The pattern of a stronger association between BMI and CHD among smokers compared with non-smokers was broadly consistent across the age, sex and region subgroups, although the test for interaction was non-significant in each of the sub-groups examined (Figure 1). There was some suggestion that the interaction was only apparent in individuals aged > 65 years (Figure 1). When we further stratified individuals into four age groups (35–59, 60–69, 70–79 and 80–89 yrs), the effect of a 2 kg/m^2 ^increment in BMI on CHD was identical in smokers and non-smokers in the youngest age-group (HR 1.16 95% CI: 1.09 – 1.22) whereas in the other three groups, the HRs were consistently stronger in smokers compared with non-smokers (results not shown but available on request).

In the sub-sample of studies for which information on former smokers was available, there were 2513 CHD events. The excess risk for CHD associated with a 2 kg/m^2 ^higher BMI was greater in current smokers than in former smokers and never smokers: HR 1.13 (95% CI: 1.09 – 1.17) versus 1.10 (1.06 – 1.14) and 1.08 (1.04 – 1.11), respectively (p-value for interaction = 0.10).

### The association between BMI and stroke risk in current and non-smokers

**Figure 2:**
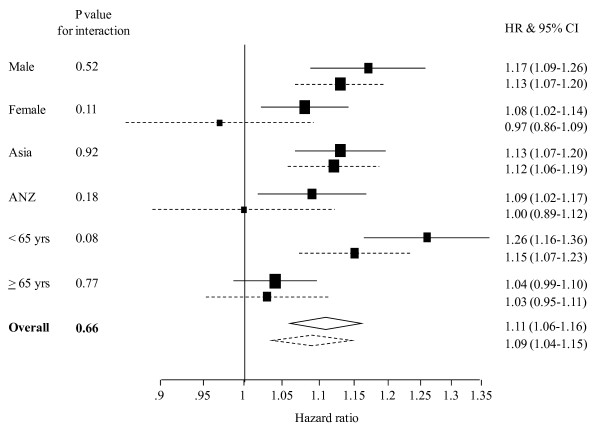
**Hazard ratios (HRs) associated with a 2 kg/m^2 ^higher body mass index for ischaemic stroke events in current smokers and non-smokers, by sex, region, age and overall**. Analyses are adjusted by age and stratified by study and sex. The horizontal lines (or widths of diamonds for overall results) show 95% confidence intervals (CIs). The p values shown are for the test of interaction between body mass index and smoking status. The dashed and continuous lines represent current smokers and non-smokers, respectively.

**Figure 3:**
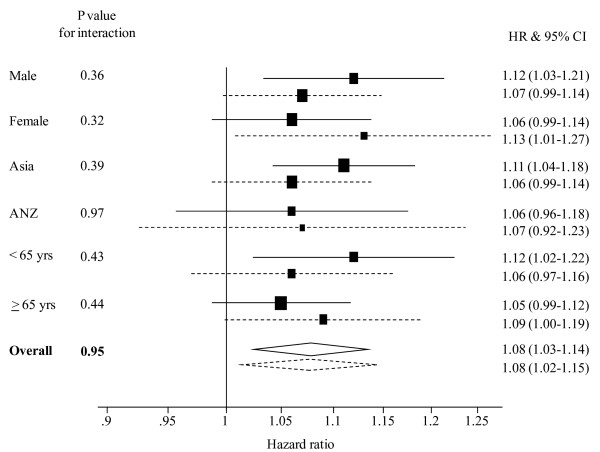
**Hazard ratios (HRs) associated with a 2 kg/m^2 ^higher body mass index for haemorrhagic stroke events in current smokers and non-smokers, by sex, region, age and overall**. Analyses are adjusted by age and stratified by study and sex. The horizontal lines (or widths of diamonds for overall results) show 95% confidence intervals (CIs). The p values shown are for the test of interaction between body mass index and smoking status. The dashed and continuous lines represent current smokers and non-smokers, respectively.

As with CHD, irrespective of smoking status, BMI was positively and log-linearly associated with the risk of ischaemic stroke, and to a lesser extent, haemorrhagic stroke and 'unclassified' stroke. When comparing the magnitude of the association between BMI and stroke risk, there was no evidence of an interaction with smoking for either ischaemic or haemorrhagic stroke: For a 2 kg/m^2 ^higher BMI, the HRs (95% CI) in smokers compared with non-smokers for ischaemic and haemorrhagic stroke were: 1.09 (1.04 – 1.15) vs 1.11 (1.06 – 1.16), p for interaction = 0.66; and 1.08 (1.02 – 1.15) vs. 1.08 (1.03 – 1.14), p for interaction = 0.95 (Figures 2, 3). In sensitivity analyses, there was no evidence to indicate an interaction between smoking and BMI by age group, sex or region for either ischaemic or haemorrhagic stroke (all p-values > 0.10). Restriction of the analyses to only fatal stroke events did not alter these findings.

**Figure 4:**
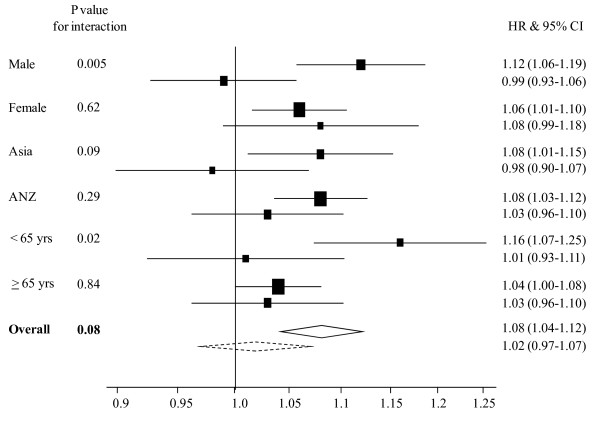
**Hazard ratios (HRs) associated with a 2 kg/m^2 ^higher body mass index for unclassified stroke events in current smokers and non-smokers, by sex, region, age and overall**. Analyses are adjusted by age and stratified by study and sex. The horizontal lines (or widths of diamonds for overall results) show 95% confidence intervals (CIs). The p values shown are for the test of interaction between body mass index and smoking status. The dashed and continuous lines represent current smokers and non-smokers, respectively.

For those strokes that were unclassified there was marginal evidence of an interaction between BMI and smoking, but in the opposing direction to that observed with CHD (Figure 4). The HRs (95% CI) associated with a 2 kg/m^2 ^higher BMI in smokers compared with non-smokers were 1.02 (0.97 – 1.07) vs 1.08 (1.04 – 1.12), p for interaction = 0.08. The apparent stronger effect of BMI on risk of unclassified stroke in non-smokers was observed in several of the subgroups. For example, in men, the HRs (95% CIs) for the risk of unclassified stroke associated with a 2 kg/m^2 ^higher BMI were 1.12 (1.06 – 1.19) for non-smokers and 0.99 (0.93 – 1.06) for smokers; p for interaction 0.005 (Figure 4).

## Discussion

This pooled analysis including information on more than 370,000 people confirms the positive log-linear relationship between BMI and CHD and stroke risk in both smokers and never smokers, with no evidence of a threshold effect within BMIs greater than 18.5 kg/m^2^. Additionally, there was some suggestion that the relationship between BMI and risk of CHD is amplified by the effects of smoking, such that the excess risk for CHD associated with being overweight in smokers was more than double that of non-smokers. There was also some evidence to indicate that the amplification effect of smoking is reversible subsequent to quitting smoking. These findings are consistent with previous work from our group, which have reported that smoking may potentiate the effects of other cardiovascular risk factors for CHD, including total cholesterol [21]. In contrast, there was no evidence to indicate that smoking amplifies the relationship between BMI with either ischaemic or haemorrhagic stroke. Anomalous to this finding was the indication that the relationship between BMI with risk of unclassified stroke may actually be stronger among non-smokers compared with current smokers. However, this observation should be treated more cautiously given the subgroup nature of this analysis, the lack of complete information on the type of stroke event and the finding of no interaction between BMI and smoking with either of the major stroke subtypes that are sure to constitute most of the unclassified strokes.

### Comparison with other studies

Earlier findings on this issue are conflicting. Although there are confirmatory data from previous studies, there are also several large studies that either reported no interaction or a slightly weaker association between BMI and CHD in smokers compared with non-smokers. For example, in a prospective cohort of 220,000 Chinese men, in whom there were 1240 deaths from ischaemic heart disease, Chen et al. observed a similar excess risk of CHD associated with 2 kg/m^2 ^increment in BMI in never and ever smokers [15]: 1.13 (95% CI: 0.98 – 1.30) and 1.12 (1.02 – 1.22) respectively; p for heterogeneity = 0.9. Similarly, in the INTERHEART case-control study there was no evidence of a significant interaction between BMI and smoking on the risk of myocardial infarction (MI) in never, former and current smokers: age and sex-adjusted odds ratios (95% CI) for MI for 1 SD increase in BMI were 1.12 (1.08 – 1.17), 1.13 (1.06 – 1.19) and 1.17 (1.11 – 1.22), respectively [22]. Whereas, in contrast to our study, the Nurses' Health Study in the Unites States [23], as well as other cohort studies from Western countries [14], observed a rather weaker positive association between BMI and CHD in current smokers compared to non-smokers or never smokers, although formal tests for interaction were not performed. For example, in the Nurses' Health Study, the relative risk for CHD comparing women with BMI ≥ 29 kg/m^2 ^to those with BMI < 21 kg/m^2 ^was 3.26 (95% CI: 2.58 – 4.12) in current smokers compared with 4.36 (95% CI: 3.12 – 6.09) in never or former smokers [23].

The reasons for these discrepant findings are unclear, but may be due to differences in the age, sex and smoking characteristics of the populations studied or, alternatively, to random noise given that the size of any interaction between BMI and smoking is likely to be relatively small. Consequently, in order to be able to reliably detect any effect it would require a sufficiently large sample size and a diversity of study populations, both are key strengths of the current analysis. Differences in how smoking is quantified between studies, length of follow-up and the suggestion of an interaction that is only apparent in men [13] may also explain some of the heterogeneity in study findings. The present study is also not without its limitations, one of which is the lack of information on changes in BMI and smoking status during follow-up. However, any potential changes, particularly those relating to smoking (for example, quitting smoking during follow-up) would have resulted in an underestimation of the synergistic relationship between BMI and smoking. Moreover, we had limited, or no, data on both the quantity of cigarettes smoked and the duration of smoking, which are both key determinants of the magnitude of the smoking hazard. Furthermore, we were limited to using BMI, a measure of general adiposity, whereas central adiposity is considered to be more closely associated with metabolic disorders than general obesity [24]. We had limited information on waist circumference and waist to hip ratio, which are better indicators of central obesity. As current smokers have been reported to have a higher abdominal fat distribution profile, in spite of their lower BMI, compared to never smokers [25,26], it may be that the effects of smoking are more apparent if indices of central obesity are used [26-28].

Excess weight is indirectly, and possibly directly, associated with CHD through its adverse effects on blood pressure, lipids and glucose metabolism [29,30]. These known coronary risk factors promote atherosclerosis of the coronary arteries in the causal pathway between adiposity and CHD. One possible pathophysiological explanation for the observed synergism is that smoking and one or more of the adiposity-induced metabolic disorders may interact to promote coronary atherosclerosis. Alternatively, smoking may affect another as yet unknown pathway between excess weight and CHD risk independent of known metabolic abnormalities.

## Conclusion

In conclusion, our data re-emphasize the importance of appropriate lifestyles and risk factor management for the prevention of CHD. Even though the amplification effect of smoking on the BMI-CHD relation is relatively small, given the widespread prevalence of smoking and excess weight (1 billion and 1.7 billion people, respectively), the population attributable risk is considerable. Thus, strategies to curb both smoking and promote weight loss at the population level could have a greater impact on reducing the burden of CHD than previously anticipated.

## Competing interests

The authors declare that they have no competing interests.

## Authors' contributions

RRH conceived the study and drafted the manuscript. FB and MW conducted the statistical analyses. KN, GDB, SC, HU, HCK, JWW XF, THL and VF provided critical revision of the manuscript. All authors read and approved the final manuscript.

## Pre-publication history

The pre-publication history for this paper can be accessed here:


